# The effect of a single injection of irinotecan on the development of enamel in the Wistar rats

**DOI:** 10.1111/jcmm.13415

**Published:** 2017-12-28

**Authors:** Sali Al‐Ansari, Rozita Jalali, Ton Bronckers, Judith Raber‐Durlacher, Richard Logan, Jan de Lange, Frederik Rozema

**Affiliations:** ^1^ Department of Oral Medicine – Academic Centre for Dentistry Amsterdam the Netherlands; ^2^ Department of Oral Cell Biology – Academic Centre for Dentistry Amsterdam Amsterdam the Netherlands; ^3^ Oral Pathology Adelaide Dental School Faculty of Health and Medical Sciences The University of Adelaide Adelaide Australia; ^4^ Department of Oral and Maxillofacial Surgery – Amsterdam Medical Centre University of Amsterdam Amsterdam the Netherlands

**Keywords:** enamel, hypomineralization, micro‐CT, mineralized tissue development, ameloblast, irinotecan, cancer chemotherapy

## Abstract

Cancer is the second most frequent cause of death in children. Because the prognosis for childhood malignancies has improved, attention has now focused on long‐term consequences of cancer treatment. The immediate effects of chemotherapy on soft tissues have been well described; however, there is less information about long‐term effects of chemotherapy on the development of dental tissues. To test the association between the effect of chemotherapy on enamel development, we examined two groups of rats: one that had received an intraperitoneal dose of 200 mg/kg of irinotecan, whereas the other (control) group had received vehicle only. Rats were killed at 6, 48 and 96 hr post‐injection; the mandibles dissected out, fixed for histological evaluation and scanned for mineralization defects by Micro‐CT. Our results showed structural changes in the ameloblast layer along with a significant reduction in mineralization and thickness of enamel at 96 hr after chemotherapy. These data demonstrate that irinotecan induces structural changes in forming enamel that become apparent after anticancer chemotherapy treatment.

## Introduction

Treatment for different types of childhood cancer has greatly improved over the past 20 years. The increasing number of long‐term survivors necessitates the importance of addressing long‐term side effects of cancer treatments. Therefore, an increasing focus has been directed towards the late sequelae of treatment modalities. A variety of late effects of childhood cancer therapy can be noted because many organs can be affected, including the oral cavity^1^, ^2^.

Late oral effects of cancer therapy are clinically significant because they have an impact on oral and general health and may negatively affect the quality of life. The most commonly observed late effects of radiotherapy to the head and neck are included reduced salivary flow and xerostomia, mucosal infections, dental caries, increased progression of periodontitis, dysphagia, trismus and bone alterations that may result in osteoradionecrosis and, in children, craniofacial and dental anomalies^3^, ^4^, ^5^. However, orofacial and dental anomalies can be also induced by anticancer chemotherapy^6^, ^7^, ^8^.

Most oral complications of cytotoxic anticancer (*e.g*. mucositis, salivary gland dysfunction, mucosal infections and taste alterations) resolve after discontinuation of therapy^9^. The extent and nature of these oral complications vary with each patient depending on the treatment modality (*i.e*. surgery, radiation therapy and/or chemotherapy) and patient‐related factors including genetic factors, age, oral health status and type of malignancy (WHO 1979). For chemotherapy, toxicity also depends on the type of antineoplastic agent(s) and the therapeutic regimen as well as the dose and duration of the treatment. In contrast to the acute oral complications, dental abnormalities and orofacial growth disturbances induced by chemotherapy for paediatric malignancies are permanent^6^, ^7^, ^9^, ^10^, ^11^, ^12^, ^13^.

Irinotecan is a drug with antitumour activity. It works by inhibiting topoisomerase I blocking the DNA replication step of the enzyme, leading to multiple single‐strand DNA breaks, which eventually block cell division^14^. Irinotecan is given against colorectal cancer and paediatric brain tumours (*e.g*. central nervous system tumours, including recurrent glioblastoma rhabdomyosarcoma and other sarcomas)^15^, ^16^, ^17^.

Dental development starts from approximately the sixth week *in utero* through late adolescence. This occurs in two phases: first, the *secretory phase* during which secretory ameloblasts deposit a partially mineralized (30%) enamel. In the second phase or *maturation phase* (when enamel reaches its final thickness), ameloblasts continue depositing calcium phosphate minerals coincident with the removal of organic material and water to attain greater than 96% mineral content. Disrupting matrix formation results in enamel hypoplasia (thinner layers of enamel), whilst reduced mineral transport by ameloblasts can cause incomplete mineralization of enamel that easily erodes after eruption. Given the fact that the permanent dentition develops from intrauterine stages until approximately age 20 (eruption of wisdom teeth), all children receiving chemotherapy are potentially at risk of developing dental abnormalities. The type of abnormality (partial agenesis, changes in tooth shape and size, altered enamel thickness, hypoplasia, hypomineralization) depends on the developmental stage at the time of treatment.

In this study, the mandibular incisor of the rat was chosen to study enamel formation as rats have continuously growing maxillary and mandibular incisors. This is characteristic for most rodents and means that throughout the animal's lifespan all stages of amelogenesis can be studied. The use of continuously forming incisors in the rodent model could show the effect of a single drug administration on each stage of enamel formation.

In this study, we tested the effect of irinotecan on the enamel development in rat incisors. We hypothesize that enamel abnormalities will become apparent after irinotecan chemotherapy treatment.

## Materials and Methods

The study was approved by the Animal Ethics Committees of the Institute of Medical and Veterinary Sciences of the University of Adelaide and complied with the National Health and Medical Research Council (Australia) Code of Practice for Animal Care in Research and Training.

In this study, archived samples of eighteen female Wistar rats, aged between 6‐8 weeks and weighing 150‐170 grams, were used. The rats were divided into vehicle control (*n* = 9) and irinotecan‐treated experimental groups (*n* = 9). The irinotecan‐treated rats received a single intraperitoneal (IP) dose of 200 mg/kg of irinotecan based on our previous work^18^, and the work of Kurita *et al*.^19^ who showed serum concentrations at different time intervals. For activation of the drug, irinotecan (supplied by Pfizer, Kalamazoo, USA) was prepared in a sorbitol/lactic acid buffer (45 mg/ml sorbitol, 0.9 mg/ml lactic acid, pH 3.4). Control group rats received an IP injection of the sorbitol/lactic acid buffer.

Control and experimental rats were anaesthetized (2% isofluorane in 100% O_2_) and killed by cardiac puncture and cervical dislocation at either 6 hr (three treatment rats and three rats control), 48 hr (three treatment rats and three control rats) or 96 hr (three treatment rats and three control rats) following administration of irinotecan or vehicle. The mandible was dissected out, and samples were fixed in 10% neutral buffered formalin before being processed. Jaw tissue was first decalcified using the Warshawsky protocol^20^. The right and left hemi‐mandibles of the same rat were divided and used for haematoxylin and eosin staining and Micro‐CT.

### Haematoxylin and eosin staining

The mandible samples were paraffin embedded, cut into 7‐μm sections and mounted onto glass slides. Sections were dewaxed in xylene, rehydrated in a descending series of ethanol, rinsed in phosphate‐buffered saline (PBS) and stained with Lillie‐Mayer's haematoxylin (Fronine Laboratory Supplier) for 10 min. Sections were counterstained with eosin (H&E staining). Images were captured at 40× magnification.

### Micro‐CT

Rat mandibles were scanned with μCT 40 (Scanco Medical AG, Bruttisellen, Switzerland). This scanner is calibrated weekly using a phantom with densities from 0 to 3000 mg HA/cm3. During the scanning procedure, the mandibles were kept wet in a cylindrical specimen holder (36‐mm) containing tap water. The integration time was set at 300 ms, the beam intensity at 55 kV, the current at 145 μA and the resolution set at 18μ. Three‐dimensional (3D) reconstructions were made using the cone‐beam reconstruction algorithm. Using two‐dimensional horizontal cross sections, mean mineral density was measured at three circular regions of interest. The shape of the surrounding bone and the position of the molar roots were used as landmarks to obtain the same stages of development for different animals. Voxels near the enamel surface were excluded to preclude micro‐CT partial volume effect. Density measurements (expressed as mg hydroxyapatite/ccm3) were made at 540‐micrometre (0.5‐mm) intervals and plotted for each tooth individually (slice number). The thickness of the incisor enamel layer measured from the perpendicular distance from early secretory until late maturation enamel and from the dentino‐enamel junction (DEJ) to the enamel surface using ImageJ software (ImageJ 1.33u software; http://rsb.info.nih.gov/ij/index.html).

### Statistical analysis

All values are presented as means ± standard deviation (SD). Data were analysed using an unpaired Student's *t*‐test. *P* < 0.05 was considered statistically significant.

## Results

### Ameloblast morphology

As shown by H&E staining, ameloblasts treated with irinotecan (96 hr) lost polarity and became short and round in the maturation stage. This is in contrast to well‐polarized ameloblasts formed in control rats. These changes started from the mid‐secretory stage and continued until the end of the maturation stage. The ameloblast layer in the other treatment groups (6 and 48 hr) was the same as observed in the control groups (data not shown). At 96 hr, the enamel matrix was homogeneous in both treated and control rats. The cellular organization of the odontoblasts and the structure of the dentin layer in the drug‐injected group were indistinguishable from control groups at all time‐points investigated (data not shown).

### Enamel mineral content

**Figure 1:**
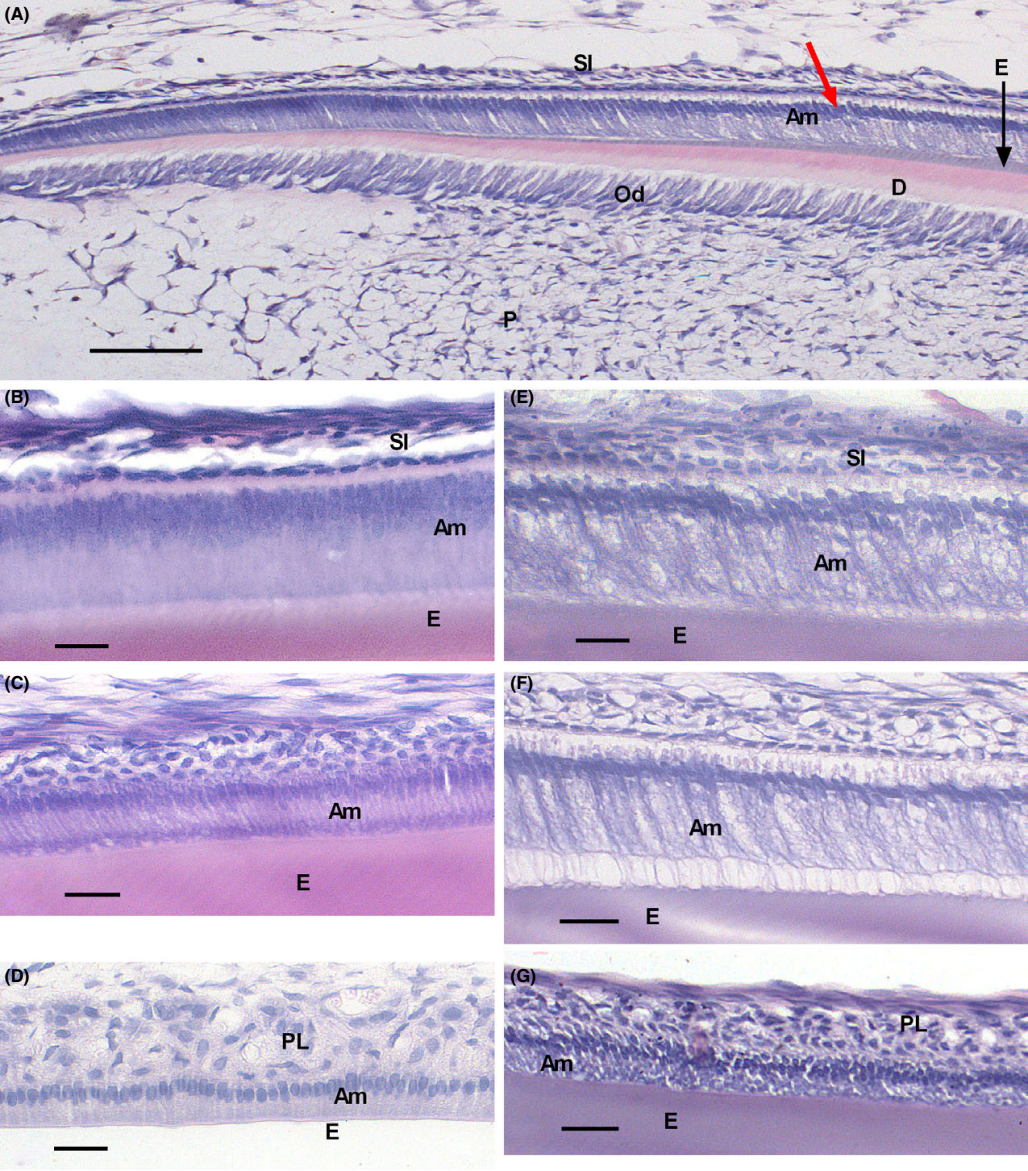
H&E staining of developing rat mandibular incisors in controls (**b‐d**) and 96‐hr chemotherapy **(A, E‐G)** groups. High‐magnification (×40) images of ameloblasts at secretory stage **(A,B,E)**, early maturation **(C,F)** and late maturation **(D,G)** of enamel development. (**A)** presents the sequence of developmental stages from the cervical end (left) to the incisal end (right) of the rat lower incisor. **(B‐D)** represents the secretory, early maturation and late maturation stages in the control group, and **(E‐G)** shows the same stages in the experimental group. Am= ameloblast; D = dentin; E = enamel; Od = odontoblasts; *P* = pulp; PL = papillary layer; SI = stratum intermedium. Note the disorganization of the ameloblasts in the experimental incisors. Scale bars: 50 μm

**Figure 2:**
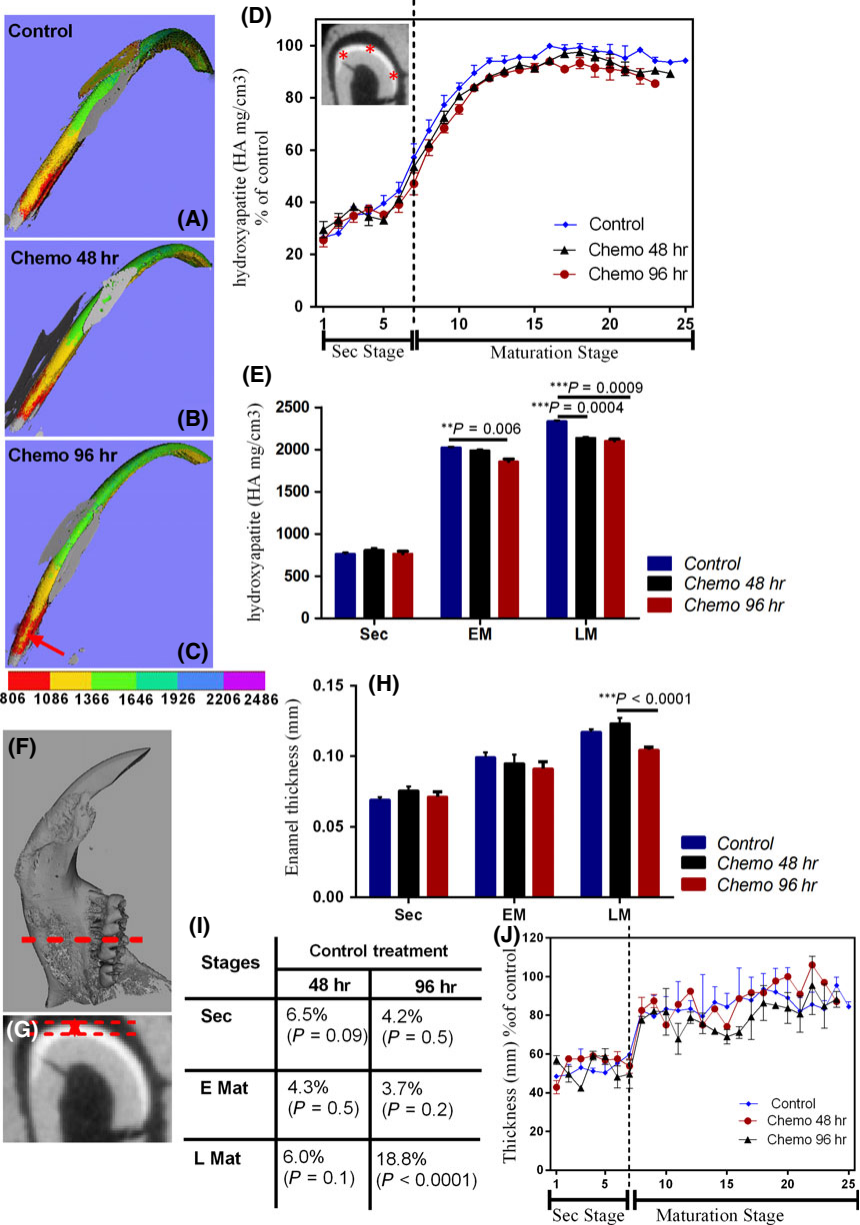
Effect of a single injection of irinotecan on mineral density and enamel thickness in rats. (**A–C**) shows the 3D reconstruction of the mandibular incisor in control group, 48 hr post‐injection and 96 hr post‐injection, respectively; different colours in (**A–C**) represent the mineral densities. In (**D)**, the effect of irinotecan on mineralization becomes apparent when mineral density is plotted as a function of development. The dotted line in (**D)** marks the transition of secretory into transitional stage enamel. Stars in (**D)** represent the points of density measurement in the enamel as shown in (**F**). **(E)** presents the average values of mineral density per group for secretory, early and late maturation enamel. The shortest distance from enamel surface to dentino‐enamel junction (line in **G**) represents the enamel thickness. These measurements were made in consecutive slides at 500‐micrometre intervals. (**I)** presents the effect of irinotecan on enamel thickness as percentage of control, (**H**)as millimetre (sec, secretory; E Mat, early maturation; L Mat, late maturation). **(J)** shows thickness reduction at 6, 48, 96 hr after injection at different stages of the enamel formation (Sec stage, secretory stage and maturation stage) (Chemo 48 hr, 48 hr after injection of irinotecan; Chemo 96 hr, 96 hr after injection of irinotecan).

To determine the effect of chemotherapy on enamel mineral content, all teeth were scanned using Micro‐CT and reconstructed in 3D. Mineralization density values were calculated for secretory stage and early and late maturation stages separately. In comparison with the control group, the enamel of the 96‐hr group was significantly less mineralized in both early maturation and late maturation stages (8.5% and 10.6%, respectively).Fig. 1 Although these mineralization differences were apparent in the 3D reconstructed images (red arrow in Fig. 2C), the values did not reach statistically significant difference (Fig. 2E Sec). The mineral density values in the 48‐hr experimental group were also lower, but not statistically significant (Fig 2E). In the late maturation stage, enamel mineral density was significantly less in both the 48‐hr and 96‐hr group as compared to controls (reduced by 9.0%, *P* = 0.004; 10.6%, *P* = 0.009, respectively). No difference was observed between the experimental and control groups at 6 hr (not shown).

### Enamel thickness

The table in Figure 2I shows thickness reduction at 6, 48 or 96 hr after injection at different stages of enamel formation (secretory, early maturation and late maturation).

Our results indicated that there were significant thickness reductions of the enamel layer (Fig. 2H,I) between the control and 96‐hr treatment group in the late maturation stage (18.8%, *P* < 0.0001).

There were no significant differences between controls and the 48‐hr treatment group at the late maturation stage (reduction of 6.0%, *P* = 0.1). No difference was observed between the experimental and control groups at 6 hr (not shown). Figure 2 presents a summary of the changes observed in the experimental groups.

## Discussion

Our results show that amelogenesis in rats is negatively affected by the administration of a single dose of irinotecan. Changes include formation of a thinner and less mineralized enamel layer which is most apparent 96 hr (see Table 1) after irinotecan administration.

**Table 1:** Summary of changes observed in enamel formation of irinotecan

	Stage	48 hr Post‐injection	96 hr Post‐injection
Enamel width	Secretion	−6.5% (*P* = 0.09)	−4.2% (*P* = 0.5)
	Early maturation	−4.3% (*P* = 0.5)	−3.7% (*P* = 0.2)
	Late maturation	−6.0% (*P* = 0.1)	−18.8% (*P* < 0.0001)
Mineral density	Secretion	+5.9% (*P* = 0.2)	−0.1% (*P* = 0.9)
	Early maturation	−1.8% (*P* = 0.06)	−8.5% (*P* = 0.006)
	Late maturation	−9.0% (*P* = 0.004)	−10.6% (*P* = 0.009)
Structural changes ameloblasts	Secretion	No	Mid secretion
	Early maturation	No	Yes round
	Late maturation	No	Yes round

Irinotecan topoisomerase I – a blocker of DNA replication – leads to single‐strand DNA breaks, ultimately blocking cell division. The drug can also affect RNA transcription resulting in less protein production. Ameloblasts at the secretory stage create the entire enamel thickness by gradual and continuous apposition of proteins at their apical plasma membrane^21^. The observation that the enamel layer in irinotecan‐injected rats is thinner than in controls can be explained by the inhibition of RNA transcription of matrix proteins during the secretion phase. The reduction in mineral density of the enamel in the experimental rats may be the result of reduced production of mineral–ion‐transporting proteins and/or their incorporation in the plasma membranes.

The results suggest that the defects induced by irinotecan cumulate over time after injection. The half‐life plasma value of irinotecan injected intravenously is 25 min in rats^22^, and 6–12 hrs in humans^23^. From the rapid clearance of the drug, it is conceivable to assume that the drug will influence the ameloblasts directly during the first hours after injection when plasma levels are highest. Effects later than 2 days when most of the drug has been cleared from the plasma are likely secondary. The strongest changes of irinotecan on ameloblast structure, enamel thinning and mineralization were seen 96 hr post‐injection in late maturation stage. In controls, after 96 hr a major portion of secretory ameloblasts has turned into early maturation cells, whereas early maturation cells had progressed into late maturation cells. Since in the chemotherapy‐treated rats, the effects became stronger with time and were strongest at the late maturation stage, and this cumulative effect of cell damage is likely due to a carry‐over of cell damage from cells treated and damaged 96 hr before. Increased defects with time suggest that ameloblasts damaged at the secretory stage cannot transform normally into functional maturation stages.

Dentin and odontoblast structure were not affected by the drug directly, only by ameloblasts. After injection of irinotecan, high levels of the drug are found in the rat liver, bile ducts and the kidney, whereas in other tissues only low levels are present. Irinotecan seems to specifically target ameloblasts, an epithelial cell type with many ion transporters similarly to liver and kidney.

These findings seem to be comparable to previous research on dental abnormalities after treatment with other cytostatic drugs^9^, ^24^, ^25^, ^26^. Dental developmental anomalies, increasing the need for dental prevention and interventions, have been reported in patients diagnosed with acute lymphoid leukaemia. Defects include tooth and root agenesis, root thinning and shortening, and localized enamel defects^9^, ^26^. The results of this study are in line with reports of changes in ameloblast morphology and function induced by other types of chemotherapy, accounting for enamel hypoplasia and increased risk of dental caries in patients with paediatric cancer^24^. Considering these risk factors, paediatric oncologists, general and paediatric dentists, and parents should be aware of these late effects of cancer treatment to secure early diagnosis and appropriate dental care. This will improve dental health and quality of life of a rapidly increasing group of survivors of childhood cancers. Further work is required to unravel the mechanisms of damage to ameloblast function and to determine the effects of other chemotherapy drugs on tooth development (Table 1).

## Conflict of interest statement

The authors have no conflict of interest related to this manuscript.
